# Understanding the care and support needs of older people: a scoping review and categorisation using the WHO international classification of functioning, disability and health framework (ICF)

**DOI:** 10.1186/s12877-019-1189-9

**Published:** 2019-07-22

**Authors:** Sarah Abdi, Alice Spann, Jacinta Borilovic, Luc de Witte, Mark Hawley

**Affiliations:** 1 Centre for Assistive Technology and Connected Healthcare, School of Health and Related Research, The Innovation Centre, The University of Sheffield, 217 Portobello, Sheffield, S1 4DP UK; 2 Aging and Health Research Unit, Faculty of Health sciences, the University of Sydney, 75 East Street, J block, Lidcombe, NSW 2141 Australia

**Keywords:** Older people, Care and support, Needs, ICF, Scoping review

## Abstract

**Background:**

The number of older people with unmet care and support needs is increasing substantially due to the challenges facing the formal and informal care system in the United Kingdom. Addressing these unmet needs is becoming one of the urgent public health priorities. In order to develop effective solutions to address some of these needs, it is important first to understand the care and support needs of older people.

**Methods:**

A scoping review was conducted, using the Arksey and O’Malley original and enhanced framework, to understand the care and support needs of older people, focusing on those living at home with chronic conditions in the UK. The search was conducted using five electronic data bases, grey literature and reference list checks. The WHO International Classification of Functioning, Disability and Health (ICF) framework was used to analyse and categorise the literature findings.

**Results:**

Forty studies were included in the final analysis- 32 from academic literature and 8 from grey literature. The review highlighted that older adults faced a range of physical, social and psychological challenges due to living with chronic conditions and required care and support in three main areas: 1) social activities and relationships; 2) psychological health; and 3) activities related to mobility, self-care and domestic life. The review also highlighted that many older people demonstrated a desire to cope with their illness and maintain independence, however, environmental factors interfered with these efforts including: 1) lack of professional advice on self-care strategies; 2) poor communication and coordination of services; and 3) lack of information on services such as care pathways. A gap in the knowledge was also identified about the care and support needs of two groups within the older population: 1) older workers; and 2) older carers.

**Conclusions:**

The review highlighted that older people living with chronic conditions have unmet care needs related to their physical and psychological health, social life, as well as the environment in which they live and interact. Findings of this review also emphasized the importance of developing care models and support services based around the needs of older people.

**Electronic supplementary material:**

The online version of this article (10.1186/s12877-019-1189-9) contains supplementary material, which is available to authorized users.

## Background

Recent statistics estimated that people aged 65 and over in the United Kingdom are expected to live almost 50% of their remaining lives with a limiting long-term physical or mental health condition [1], thus increasing their need for care and support. Indeed, around 20% of men and 30% of women in this age group currently need help with at least one Activity of Daily Living (ADL) [2]. These numbers are likely to increase in the future; current predictions suggest that by the year 2035, the absolute number of older adults with low or high dependency will increase by almost a third [3], raising a significant challenge to meet their needs for care and support.

It is now well acknowledged that the health and social care system in the UK is struggling, and to a certain extent failing, to meet the care and support needs of older adults [4–7]. A recent analysis of data from wave 7 of the English Longitudinal Study of Ageing (ELSA) revealed that 50% of older people who have difficulty with an ADL received no formal or informal support [8]. There is also a growing concern about the ‘unnecessary’ time spent by older adults in hospitals due to delayed discharges [6, 9], which can lead to worsening their health outcomes and complicating their care and support needs. These situations are likely to be exacerbated in the future, given the increasing funding pressure and the steep decrease in the health and social care workforce [10]. Additionally, and due to the challenges in the formal care system, the number of unpaid carers has been growing fast contributing to almost two third of the provided care [4, 5]. Although the role played by carers is integral to older adults and the care system, the significant impact caring has on their physical and mental health as well as on their finances raises questions about the long-term sustainability of unpaid care [11]. Collectively, it is evident that there is a clear challenge to meet the care and support needs of an ageing population both now and in the future.

Addressing the unmet care and support needs of an ageing population, and designing services and solutions centred around what older people need or want, is becoming an urgent public health priority [6, 8]. In order to address those needs effectively, it is important first to identify and understand the care and support needs from the perspective of older people as well as understand the wider context in which they live and interact. To date there is limited recent evidence synthesis regarding the care and support needs of older people living with chronic conditions in the UK. In a systematic review investigating the impact of multimorbidity on older people, Marengoni and colleagues [12] reported that functional decline, poor quality of life and high healthcare costs are amongst the major consequences of living with multi-morbid conditions. However, limited information was provided in the review on the type of support required by older people to cope with these challenges. Similarly, Young and Tinker [13] investigated recently the future needs and preferences of older adults in the UK, however, the review didn’t report needs within the area of care and support and was focused on a particular group within older people (1960 baby boomers). In a more recent review, McGilton and colleagues [14] reported several areas of needs for older people with multiple conditions, highlighting poor coordination of services and lack of information as areas of prominent needs. Nevertheless, a large proportion of the studies in this review were based in North America, with some evidence coming from the UK. Arguably, although there are some similarities between the UK and other western countries in the health and social care challenges faced, there is still a need to provide a more in-depth analysis of the care and support needs of older people in the UK. This owes to the fact that the care and support required by older people depends largely on the services and support available or provided to them, which are influenced in many cases by country-specific challenges. Therefore, a scoping review was conducted to identify and understand the care and support needs of older people in the United Kingdom, focusing on those living at home with chronic conditions.

## Methods

A scoping review was conducted to systematically scope and synthesise the evidence on the care and support needs of older people living at home in the United Kingdom. A scoping review design was deemed appropriate as this approach allows to systematically examine the literature and summarise the findings in a particular area of study, identify gaps in the existing knowledge, as well as refine the search strategy when new information emerges and a deeper knowledge of the literature and the key concepts are gained [15–17]. The scoping review design was developed based on the Arksey and O’Malley original and enhanced framework for conducting a scoping review [15, 16]. This framework recommends six steps in conducting a scoping review: 1) identifying the research question; 2) identifying relevant studies; 3) selecting the studies; 4) charting the data; 5) organising, summarizing and reporting the findings; and 6) stakeholder consultation (optional). The following sub-sections report the methods used to conduct step one to five.

### Identifying the research question

This review aimed at answering the following research question “What is known from the existing literature about the care and support needs of older adults living at home with chronic conditions in the United Kingdom?”. The focus of this review was chosen as older adults living with chronic conditions, since the care and support needs arise largely from disabilities, physical or mental impairment or illness [18]. Also, this review aimed to focus on older adults living at home, given that the majority of older adults in the UK live in their own homes [2], with many preferring to remain and continue living in their homes as long as possible [19, 20]. Supporting older adults to continue living in their own homes is also a priority to many local authorities in the UK [5].

### Identifying relevant studies

A scoping search was first conducted in MEDLINE via Ovid and Applied Social Sciences Index and Abstracts (ASSIA) to gain familiarity with the topic and the volume of the literature. The initial search terms for the scoping search were developed to reflect the key concept areas addressed by the research question. These areas were: ‘needs for care and support’, ‘older adults’ and ‘chronic conditions’. The search terms were revised based on the search results to ensure that key terms were included in the final search. Advice was also sought from a social care expert, two librarians and an information specialist to ensure that the search strategy was in line with the research question. The final search strategy was first piloted on Medline via Ovid and then translated to the remaining databases which included: PsychInfo via Ovid, Cumulative Index to Nursing and Allied Health Literature (CINAHL), Applied Social Sciences Index and Abstracts (ASSIA) and Google Scholar. Additionally, Social Care Online (from the Social Care Institute for Excellence) was used to identify articles for this review. Table 1 outlines the final strategy on Medline via Ovid.

**Table 1:** Final search strategy on Medline via Ovid

	Key concepts		Search terms
	Needs for care and support	OR	“care and support”.ti,ab., “care need*”.ti,ab., “health need*”.ti,ab., “social need*”.ti,ab., “patient* need*”.ti,ab., “unmet need*”.ti,ab., “lived experience*”.ti,ab, “health priorit*”.ti,ab., “care preference”.ti,ab., “activit* of daily living”.ti,ab., Health Service Needs/, Patient Preference/
AND	Older adults	OR	“older adult*”.ti,ab., “older people”.ti,ab, elderly.ti,ab., elders.ti,ab., “ageing population*”.ti,ab.
AND	Chronic conditions	OR	“chronic disease*”.ab,ti,sh., “chronic condition*”.ab,ti,sh., “long-term illness*”.ti,ab,sh., “long-term condition*”.ti,ab,sh., “long-term disease*”.ti,ab,sh., disabilit*.ab,ti,sh., multimorbid*.ab,ti,sh., “multiple chronic”.ti,ab,sh., “multiple morbid”.ti,ab,sh.,Chronic, Illness/, Comorbidity/, Long Term Care/

* This symbol represents unlimited searches for variations on a word that are formed with different suffixes

Additionally, the reference lists of the articles included in the final analysis were checked to identify additional relevant references and ensure that no key articles were missed. Grey literature was also examined via searching the websites of key national health and social care organisations. These included: Age UK, Centre for Ageing Better, King’s Funds, Nuffield Trust, 102 NHS, Department of Health and Social Care and Public Health England.

### Selecting the studies

This stage involved selecting the articles in three steps: 1) title screening; 2) abstract screening; and 3) full-article screening. Studies were selected as per inclusion and exclusion criteria that were developed based on the Population, Concept and Context (PCC) framework [17]. In brief, studies were selected if they: 1) included older adults with chronic conditions (population); 2) described their care and support needs (concept); and 3) included older adults living at home in the United Kingdom (context). In the title scoring phase, the aim was to obtain an overview of the extent of research in the area of care and support and older adults, hence a broader scope was taken where no limitation was applied to the context (the country and the home setting criteria). These two criteria were added at the abstract stage where articles not focusing on older adults living at home in the UK were excluded from the subsequent screening. Articles were also excluded if they were not in English or full text was not available. The publication date was limited to articles from January 2008 to May 2018 to ensure that findings reflect current and potential future needs of older adults living at home in the UK. Grey literature resources were also screened based on these criteria. Table 2 provides further details on the inclusion and exclusion criteria used in this review.

**Table 2:** Inclusion and exclusion criteria based on the Population, Concept and Context (PCC) framework

	Inclusion criteria	Exclusion criteria
Population Older adults with chronic conditions	Studies were included if they: • Describe the perspectives of older adults with chronic conditions. ο If the study doesn’t mention older adults explicitly, older adults were defined as adults aged 65 or above^a^. ο Chronic condition was defined as ‘conditions that cannot, at present, be cured but is controlled by medication and/or other treatment/therapies’^b^.	Focus exclusively on: • Other age groups (eg. Children) or caregivers or health and social care professionals or any other group *OR* • Acute conditions
Concept Need for care and support	Studies were included if they: • Discuss care and support needs of older adults. This was defined as ‘tasks or challenges faced by older adults in their daily lives that are related to their physical and/or mental illness or condition, and for which they need/want external assistance or support*.’ OR* • Describe the lived experience of older adults with chronic conditions *OR* • Describe care seeking behaviours of older adults in emergency or primary care	Focus exclusively on: • The prevalence/incidence of a chronic condition *OR* • Disease diagnosis/ aetiology/ clinical management *OR* • Development, evaluation or assessment of interventions, services or clinical tools *OR* • Determinants of health such as income, social status, education level, employment, genetics, gender, race, biomarkers. *OR* • Financial needs such as housing benefits or pension credit
Context Living at home in the United Kingdom	Studies were included if they: • Are based in the United Kingdom. *AND* • Include participants living in their own homes.	Focus exclusively on: • Older adults’ experiences in care and residential homes, inpatient clinics or hospitals or other settings such as prison. *OR* • Non-UK setting.

^a^Based on NICE definition of older adults: “people aged 65 or older”. [21]

^b^Long term conditions can include: “physical and mental health conditions, complex symptoms like pain or frailty, sensory impairment such as hearing or sight loss, or ongoing condition such as learning disability.” [22] Falls and fractures were included in this category as they are usually associated with several chronic conditions (osteoarthritis, frailty, cardiovascular problems etc.) and can lead to prolonged need for care and support [23]

The title and the abstract screening were conducted by two independent reviewers (SA and AS). A scoring system was also developed to approach the screening of articles systematically, where an article was given a score of two if it met the inclusion criteria fully, one if the reviewer was not sure about its eligibility, and zero if it failed to meet the inclusion criteria. The scores of both reviewers were then summed and titles with a score of two points or more were screened in the abstract screening phase. The same process was repeated in the abstract screening phase, and articles with a score of two points and more were included in the full article screening phase. Significant disagreements between the 1st and 2nd reviewer, where one of the reviewers scored an article as 2 and the other reviewer scored it as 0, was resolved by discussion and seeking opinion from a third reviewer. Full articles and grey literature resources were screened by SA and an opinion from a second reviewer was sought in case of uncertainty. Cohen’s Kappa was calculated to determine inter-rater reliability [24, 25].

### Charting the data

Data from articles and grey literature resources judged to have met all inclusion criteria was charted using a data charting form on Microsoft Excel. The form was developed by the primary author (SA) to capture information relevant to the research question. The form was then piloted by two reviewers (SA, and JB) using five articles. The final form included the following information: Author(s), year of publication, study objective(s), study location, study design, the chronic condition(s) under investigation, sample size, methods of recruitment and data collection, inclusion and exclusion criteria, participants’ characteristics, main results related to the care and support needs of older adults. Additional information was charted for the grey literature and it included: the name of the issuing organisation and the type of document.

### Organising, summarizing and reporting the findings

The charted data from published articles and grey literature were analysed using two main strategies: 1) a descriptive numerical summary highlighting the main characteristics of the studies and 2) a qualitative thematic synthesis. The methods used to analyse findings thematically was guided by Thomas and Harden [26] approach that describes three stages of conducting qualitative thematic synthesis (coding text, developing descriptive themes and developing analytical themes), and was mainly conducted deductively using the WHO International Classification of Functioning, Disability and Health (ICF) framework [27]. The ICF is an international framework used to describe and classify information related to health, disability and functioning and is underpinned by the concept that someone’s level of functioning and disability is a result of interactions between their health condition, environmental factors and personal factors [27]. Using the ICF for data analysis was reported to facilitate the comparison of data on functional status across diseases and between countries, as well as help in providing a detailed analysis of people’s experiences from their own perspective [28]. Using this framework was also useful in creating the analytical themes, a process described by Thomas and Harden [26] as controversial and often difficult to describe. Many studies that used ICF for data analysis followed an inductive approach and then linked the themes to the ICF component [28]. This approach was found appropriate for this review, since using an inductive approach prior to the use of ICF framework might have overcome some of the limitations associated with using standard frameworks for qualitative synthesis. The process of analysis is described in the following steps:

1. At the start of the analysis, papers were read multiple times to get familiar with the data and plan the coding phase. All papers were then imported into NVivo software (QSR International, 2018) to facilitate coding of the data.
2. The findings sections of the included studies were then coded line-by-line, labelling text with codes that thought to describe the content and the meaning of the text. The findings section was taken to be the text under the ‘results’ or ‘findings’ section as well as quotations from participants. In articles that included older people as part of the sample, the care needs of older adults were identified mainly from quotations from older participants, as well as from texts indicating that findings are from older people. The coded text varied from short phrases to large amount of text.
3. The initial codes developed were then transferred to subsequent studies, creating new ones when necessary. After completing this step of analysis, all codes and attached text were revised to ensure consistency of interpretation and to check whether additional coding was required.
4. The codes were then reviewed for similarities, differences and relationships, and were sorted into preliminary themes. This step also involved collating relevant coded data extracts within each of the preliminary themes. The initial codes and preliminary themes were developed iteratively by the first author (SA) and were discussed within the research team to ensure they reflected the analysed data.
5. After the initial themes were identified, the analysis was conducted deductively, in which the themes were compared and matched with the International Classification of Functioning, Disability and Health (ICF) framework. Thus, some of the themes were joined or divided in order to align them with the ICF classifications.
6. The themes and sub-themes were then named and defined based on the ICF definitions.
7. A summary of each theme was written and checked against coded data extracts and full articles to ensure accuracy.

## Results

### Summary of the literature search

**Fig. 1:**
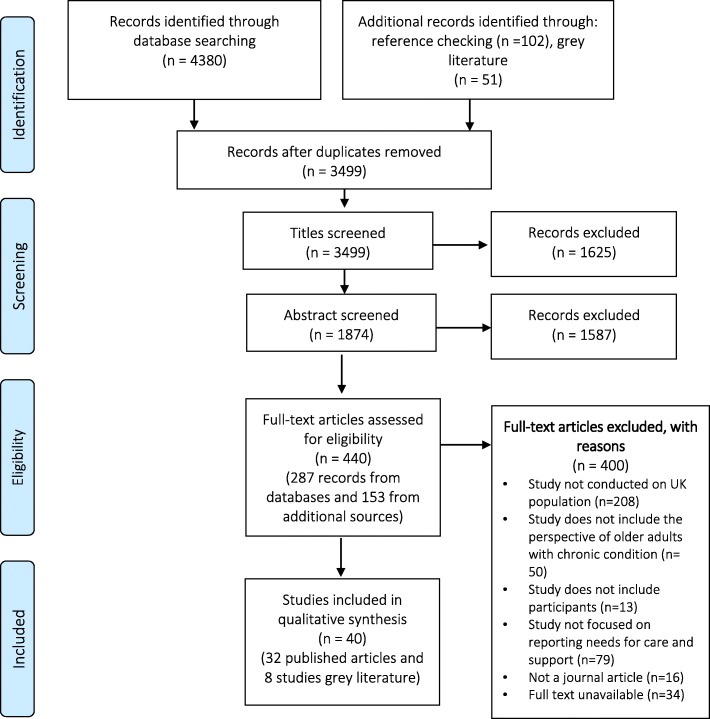
PRISMA 2009 flow diagram showing the numbers of publications identified and screened for eligibility during the scoping review (insert after the literature search in the results section, page. 10)

The electronic searches of databases resulted in 4380 records. After removing duplicates, 3499 titles were screened for eligibility. The scoring and selection of titles resulted in 1874 records that met the inclusion and exclusion criteria. The abstracts of these records were then screened, resulting in 287 records for full-text assessment. Additionally, 153 texts were identified from other sources and were screened for eligibility (102 through reference checking, and 51 from grey literature resources). Following the exclusion of studies that did not meet the inclusion criteria, a total of 40 studies were included in the final qualitative analysis (see Fig. 1. PRISMA flowchart). The Cohen Kappa for agreement between the two reviewers was 0.56 in the title scoring stage and 0.57 in the abstract scoring phase, which is considered moderate agreement.

### Characteristics of studies

#### Study objectives and designs

40 studies were included in the final analysis, of which 32 were published articles [29–60] and 8 were studies identified from grey literature [61–68]. Of the 32 published articles, 13 aimed at exploring participants’ general needs for care and support and/or reported their experience of living with chronic conditions [29–41, 52], while the remaining articles focused either on certain aspects of participants’ living experience [42–51] such as management of the condition [42–46] or on a specific period of participants’ lives [53–60] such as end of life [53–57]. Three of the grey literature studies aimed at exploring the lived experience of older adults [61, 62, 67], while the remaining studies [63–66, 68] focused on older adults’ views of specific services such as home care [63], transport services [65], and home adaptations [66]. The total number of participants in the published articles was 7871 ranging from 7 [32] to 4886 [50] and distributed across 25 qualitative studies (*n* = 820) [30–35, 37–45, 47, 48, 51, 52, 54–56, 58–60], 6 quantitative cross-sectional or survey studies (*n* = 7051) [29, 36, 49, 50, 53, 57], and 1 mixed methods study (*n* = 18) [46]. Of the eight grey literature studies identified, four used qualitative methods [61, 62, 64, 66] and included a total of 133 participants, two were of mixed methods design and included a total of 2455 participants [67, 68], whereas the remaining two were summary reports based on data from surveys, focus groups and case studies [63, 65].

#### Participants’ characteristics

The mean age of participants ranged from 64.9 [42] to 89.9 [31] in the published articles and from 75 [64] to 84 [62] in grey literature studies. The percentage of female participants ranged from 14% [43] to 92% [35] in published articles and from 53% [63] to 80% [61] in grey literature resources. Twenty-three published articles focused exclusively on older adults or on conditions associated with old age, with dementia being the most frequently studied condition [30, 34, 35, 43, 44, 47–49]. In the remaining published articles, older adults were included as part of the sample, with chronic obstructive pulmonary disease (COPD) and breathlessness being the most frequently investigated condition [39, 40, 54, 55]. Two of the grey literature studies focused exclusively on older adults with frailty [61, 62], whereas the remaining studies included older adults with a range of chronic conditions [63–68]. Three studies only investigated the impact of multi-morbidities on participants’ lives [34, 38, 48], with two of these studies reporting the experience of participants with dementia and a concurrent condition such as visual impairment [34, 48]. In terms of ethnicity, White British comprised 78% [57] to 94% [51] of the total sample in published articles and 80% [61, 62] to 93% [66] in grey literature resources. 22 published studies focused exclusively on participants living in their own homes [29, 31, 33, 34, 37–41, 44, 45, 47–50, 52, 54–58, 60], while the remaining studies included samples from mixed living arrangements. The percentage of participants living alone in their own homes ranged from 16% [44] to 87.5% [60] in published articles and from 20% [61] to 78% [64] in grey literature resources. The majority of published studies (*n* = 25) were conducted in England, with only four studies conducted in Scotland [35, 46, 55, 60], 1 in Wales [26] and 2 studies were based on national samples [29, 50]. Four of the grey literature studies were conducted in England [61, 62, 67, 68], whereas the setting was not clear in the remaining resources [63–66]. A summary of the characteristics of the studies can be found in an additional file [See Additional file 1].

### Main findings

Three main themes were identified based on the ICF classification system: 1) Body functions; 2) Activities and participation; and 3) Environmental factors. A detailed description of the findings of each of the themes is provided in the following subsections. A list of the studies that discussed each theme grouped by conditions can be found in an additional file [See Additional file 2]. A summary of the findings of each theme can be found in an additional file [See Additional file 3].

### Body functions

This theme describes the physiological problems faced by participants in the analysed studies which include mental and physical functions.

#### Mental functions

Various experiences triggered a range of negative emotions in participants in the analysed studies. Diagnostic tests and the diagnosis process were described by many participants as stressful time due to the uncertainties associated with it [40, 43, 46]. Participants in three studies also felt that their emotional needs were not catered for by health professionals and were left to face their diagnosis alone [43, 46, 47]. Some symptoms, such as visual hallucinations [30] and breathlessness [39, 56], also left participants with negative feelings such as anxiety, worry, frustration and fear. Participants in several studies expressed fears and worries of losing independence and being burden on others, with feelings such as depression, loss of pride and emotional pain used by some participants to describe their physical losses [33, 40, 46, 59]. Uncertainty about the future, particularly in conditions with poor prognosis (heart failure, dementia, cancer, advanced COPD) also triggered negative emotions such as loss of confidence in one’s abilities, despair, anxiety and fear [37, 39, 44, 54, 56]. However, participants in some of these studies seem to attribute their poor prognosis to advancing in age rather than their conditions and only a few of them discussed their concerns with health professionals [54–56].

#### Physical functions

Several physical impairments were reported in the analysed studies. These included pain [31, 36, 37, 39, 40, 52, 53, 56, 57, 61, 63, 65, 66, 68], breathlessness [39, 40, 52, 57, 61], visual and hearing impairments [30, 34, 46–48, 58, 65], fatigability [31, 37, 40, 52, 57, 61], urinary incontinence [36, 58, 63, 66] and impaired functions related to the digestive system [36, 53, 57]. Many studies investigated the emotional, physical and social impact of visual impairment and breathlessness on participants’ daily activities [30, 34, 39, 40, 46–48]. For instance, the presence of visual impairment, was reported to exacerbate existing difficulties in managing dementia and placed significant constraints on participants’ social lives. On the other hand, a few studies only discussed the impact of pain on participants’ daily lives. Further details on the physical and social limitations faced by participants are discussed in the following themes.

### Activities and participation

This theme describes the difficulties faced by participants in performing activities related to self-care, domestic life, mobility as well as problems they encountered in involvement in social and community life. Self-care describes tasks about caring for oneself such as washing, dressing and maintaining one’s health, whereas domestic life describes domestic tasks such as household cleaning and shopping. This theme also discusses strategies used by participants to manage their own physical and mental health.

#### Self-care and domestic life

Participants in several studies reported having difficulties in carrying out self-care and domestic life tasks [29, 31, 36–40, 43–45, 47, 49, 52, 53, 58, 59, 61, 63, 66]. However, only some of these studies provided details on the tasks that were affected. Washing, dressing and toileting were the main tasks that participants reported having problems with in the studies that discussed the affected self-care tasks [29, 37–39, 49, 59, 63, 66]. Participants with breathlessness, for instance, reported having problems with bathing due to steam, or difficulty standing [39]. Similarly, participants with dementia were reported to have problems with dressing, bathing and continence from the early stages of the disease onwards [49]. In studies that reported domestic life activities, difficulties with looking after the home was commonly mentioned [37, 39, 48, 50, 52, 53], followed by shopping [37, 47, 53, 63] and preparing meals [47, 53, 61]. For instance, participants with age related macular degeneration, reported how their cooking skills were affected by their sight loss due to difficulties with simple tasks such as chopping food [47]. However, and in spite of the difficulties associated with performing domestic and self-care tasks, some participants continued to do them to maintain a sense of independence and identity, and to feel a sense of purpose [39, 52, 60–62, 66]. Providing care to others, for instance, was an activity that gave some participants a sense of purpose despite being challenging [5].

#### Mobility

Many participants also reported having problems with mobility. These included difficulties with walking [33, 37, 57, 59, 61, 66], changing body position [39, 45, 50, 63], lifting and carrying objects [38, 39], hand and arm use [50, 57]. Participants’ inability to change and maintain body positions like kneeling, bending, standing was reported to affect their abilities to perform domestic activities such as cooking, cleaning home and shopping [39, 50, 59]. Similarly, problems with arm use affected tasks such as lifting objects and dressing, in participants with breast cancer [38]. Difficulties with walking was also reported in participants with frailty [31, 33, 61], hip fractures [37, 59], and advanced Parkinson disease [57] and in some cases limited participants’ mobility outside their homes [33, 37, 61].

#### Interpersonal interactions and relationships, community and social life

Social isolation and feeling of loneliness were reported by participants in several studies [30, 31, 33, 34, 37, 39, 40, 47, 48, 52, 61, 62, 67, 68]. Some participants reported that physical impairments such as physical frailty, lack of independence, or ill-health reduced their ability to sustain relationships and hence contributed to their social isolation [33, 48, 67]. Others were unable to recognise people and/or engage in social interactions, particularly in group interactions, due to sight loss [30, 46] or hearing loss [34]. These impairments also limited participants’ abilities to enjoy hobbies and leisure activities [30, 34]. Some participants, thus, became dependent on their close relatives for daily stimulation and social interactions, increasing their feelings of boredom and social isolation in the absence of these close interactions [34, 48]. Indeed, losses of close relatives or friends were reported by many participants to contribute to their feelings of loneliness and social isolation [33, 52, 61, 62]. Some participants, in one grey literature report, also valued the opportunity that work had provided them in the past for social interactions as well as other benefits such as financial security, with some of them reporting going back to work after a period of retirement [67]. Indeed, the ability to still be able to work and contribute usefully was valued by some of the younger participants in studies that included adults aged 50 and above [32, 45, 67, 68]. However, ill-health, the stress of managing chronic conditions at work, and the lack of support in the work environment was reported, mainly in grey literature reports, to force some of them to leave their job [40, 67].

#### Managing own physical and mental health

In spite of the psychological, physical and social difficulties faced by participants, many of them used strategies to self-manage their conditions and cope with these limitations. Some of the physical adaptation strategies included pacing oneself and changing the body movement to a comfortable position to reduce knee pain or cope with frailty [31, 45, 61], attending pulmonary classes to learn how to manage breathlessness [40], using alternative therapy for hand osteoarthritis [42] and keeping physically active and watching diet [67]. Another strategy that some participants found useful was establishing a daily routine [31, 33, 37, 44, 66], although in some cases their ability to maintain this was compromised by unpredictable home care visits [33, 61]. In many cases, these strategies were developed by participants themselves through personal experimenting and without professional advice. Many participants also developed psychological strategies to overcome difficulties. These included accepting limitations caused by their conditions [30, 37, 39, 44, 47, 53, 55, 60], changing attitude towards life and being positive [44, 47, 57, 60], mental distraction and occupying time with activities [35, 39, 45], spirituality [40, 47, 61] and humour [39, 61]. Some participants, however, used strategies that might not necessarily be positive, such as denial [40, 47].

### Environmental factors

This theme discusses the social and physical factors that participants interact with, which may act as facilitators or barriers to their lives. It includes ‘support, relationships and attitude’, ‘services’ and ‘products and technology’. ‘Support and relationships’ describe the support provided to participants by their close relatives, friends, peers, professionals, community and their attitude. The ‘Services’ subtheme describes the health, social and other services that are designed to meet the needs of participants. ‘Products and technology’ describe general and specifically designed products, equipment and technology that participants used in daily living.

### Support, relationships and attitude

#### Family, friends, peers, community and their attitude

The importance of the support provided by family and friends was demonstrated in several studies [29, 30, 33, 36, 37, 44, 45, 47, 48, 51–53, 57–59, 61, 62, 65–67]. Family carers offered support to participants by finding information and coordinating services for them [48, 51], assisting them with daily activities [29, 33, 58, 59, 61], and offering them company [30, 58, 61, 67]. Participants also reported feelings of happiness, joy and pleasure when interacting with family and friends [32, 47], valued peer support as an important source of information and companionship [44, 62], and identified relationships with family and friends as the most important thing in their lives [52]. In spite of these positive contributions to their lives, some participants reported feelings of being patronised [30, 44, 48], stigmatized [40, 44], not understood by family and friends [48] and were perceived differently after a period of increased vulnerability [31]. Frequent unmet needs were also reported in areas where informal carers were the main source of support [58], with participants in one study describing care provided by family as inadequate and unreliable [61]. Concerns were also raised regarding participants who live alone who might not have access to an informal social support system [33, 58, 67]. Geographical spread of family and friends also made it difficult for some participants to keep in contact with them [33, 52].

#### Care professionals and their attitude

The role of the professional support was reported in many studies [31, 35, 38, 44, 47, 48, 51, 52, 54, 55, 59–61, 63–65]. Health professionals, particularly specialist nurses, were identified by many participants as a primary and trusted source of information [38, 51, 54, 55, 64]. Interactions with health and social care professionals was also reported to provide a source of comfort and reassurance to some participants [31, 52, 60]. However, insufficient professional support was reported in several areas [31, 33–35, 38, 40, 42, 48, 54, 56, 57, 60, 61, 63]. Many participants reported lack of information and advice by health professionals in areas such as diagnostic procedures [35], care after hospital discharge [64], management of conditions [40, 42, 48, 57] and existing co-morbidities [38, 48, 61]. For example, some participants with repeated hospital admission reported that poor quality discharge and lack of clarity on after care contributed to their hospital readmission [64]. Another area where participants reported lack of advice was on the prognosis of diseases, however, there was a preference from some participants not to seek information from health professionals about this topic [55, 56]. Consistency in having the same care professional was seen as helpful [34, 35, 63], particularly in the case of participants with dementia [34, 35], however, this was not possible in many cases due to the high turnover of staff [34, 35].

#### Services

Participants’ experiences and use of services were discussed in some studies, focusing mainly on the interactions with health and social care services [29, 30, 35, 37, 40, 44, 47, 48, 51, 53, 55, 58, 59, 61, 63–66]. Some participants expressed their satisfaction with the specialist services provided to them which included intermediate care services [37], social services [47] and unspecified specialist services [44, 61]. A few participants also reported having a positive experience of care during hospital readmission and felt that hospitals were the best place to deal with their problems [64]. However, participants’ dissatisfaction with health and social care services was reported in several instances [30, 35, 40, 44, 51, 53, 55]. For example, poor coordination and integration of services was seen as challenging by many participants [35, 40, 44, 48, 64], leading to delays in service delivery [35] and compromising the management of pre-existing conditions [48]. Participants in some studies also expressed concerns with lack of information available to them on care services and pathways [35, 44, 51, 63, 65, 66]. For instance, some participants mentioned that chance conversations with people with similar needs, and previous links to health and social care services, were their source of information about services [51], raising concerns about people without these links. The need to increase access to services such as day centres, transport and home care was also reported by some participants [35, 44, 53, 61, 63, 65]. For example, poor access to accessible, comfortable and reliable transport services as well as lack of information on these services was reported to complicate some participants’ journeys to hospitals, leading to missed appointments and negative consequences on participants’ health [65].

#### Products and technology

Some participants reported using equipment and technology to cope with physical difficulties [34, 37, 41, 42, 44, 45, 47, 52, 59–61, 66]. The use of mobility aids such as wheelchairs, walking sticks and walking frames was reported by some participants with history of falls and fractures [37, 59, 60], dementia and visual impairment [34], breathlessness [39], and frailty [61]. However, the use of these aids was not always perceived positively, with some participants refusing to use them due to seeing it as markers of loss of independence [31, 60, 61]. Visual aids were used by some participants with visual impairment, however, they reported some difficulties with their use such as being bulky, expensive and in some instances not usable due to the presence of another impairment like memory loss [34, 44, 47]. Other devices reported in the analysed studies included pendant alarms to increase participants’ safety at home [52, 61], assistive devices for hand osteoarthritis [42], and prostheses for participants with breast cancer [38]. Some of the barriers to the use of these devices included being uncomfortable [38], lack of information [41, 42], and their interference with daily lives [52]. Adapting the home environment was reported as one of the strategies used by some participants to increase indoor mobility, facilitate the use of assistive devices and to increase or sustain familiarity within home [33, 34, 41, 59, 66]. However, the cost associated with some of these adaptations, the lack of information and advice, the unattractive design of equipment and the poorly fitted equipment [41, 66] might act as potential barriers to home adaptations.

## Discussion

The aim of this review was to identify the care and support needs of older adults, focusing on those living at home with chronic conditions in the UK. Three main areas emerged from the analysis that older adults faced some difficulties with and required external support. These areas were social life, activities related to self-care, domestic life and mobility, and psychological health.

### Social life

This review highlighted the value of social relationships and social interactions to older adults. This was demonstrated in feelings of loneliness and social isolation expressed by many participants when losing the ability to sustain relationships or engage in social activities due to their illness. Poor health is acknowledged to increase the risk of social isolation and loneliness [69, 70], increasing the need for supporting older adults in this area. This review highlighted that for many older adults, family and close friends provided companionship and facilitated social and pleasurable activities. However, unmet social needs were reported by some older adults with good social contacts in this review, highlighting the fact that having a social network doesn’t necessarily combat loneliness or translate into better social connectivity. Indeed, high prevalence of loneliness was reported recently in older people living with others as well as in those living alone [71], suggesting the need to increase older adults’ access to ‘meaningful’ relationships and not only increase their social contacts. The need to distinguish between social isolation, loneliness and living alone, has been under debate recently [72, 73], identifying it as an important issue when tackling this problem in the older population.

Supporting older adults in improving their social connectivity and reducing loneliness has also been targeted by many initiatives recently [74–77]. However, and apart from few examples of the use of day services and peer support groups, there was limited evidence from the reviewed literature on older adults’ access to such support. Knowledge about the views and experiences of older adults of these support services is still evolving [71], with most recent studies focusing on the quantitative evaluation of these interventions only [76, 78–80]. Some of the barriers identified in recent qualitative work [71] included older adults feeling stigmatised by services targeting ‘lonely’ older adults, with most expressing preferences to engage in activities with a purpose [71]. However, these views came largely from an active and mobile group and might not necessarily be representative of older people who have difficulties leaving the house, as with many participants in this review.

Older adults can also develop their own strategies to cope with loneliness such as acceptance of low levels of social contacts and keeping busy with solitary activities [70]. Nevertheless, this review highlighted limited evidence on such strategies attributed perhaps to lack of older adults’ awareness or lack of professional advice on social coping strategies [81]. Collectively, it is clear from the evidence reviewed that there is a need to increase older adults’ access to support in this area and understand barriers and facilitators to access support services. There is also a need to further understand strategies used by older people to cope with social difficulties.

### Self-care, domestic life and mobility

This review highlighted that many older adults living with chronic conditions experienced difficulties with tasks related to mobility, self-care and domestic life, and were in many cases dependent on family carers and home care services to provide support. The significant role family carers have in supporting older adults to meet their needs in this area is well-recognised in the literature [4, 6]. However, concerns were also raised about the long-term sustainability of family care due to the impact caring has on carers’ physical and mental health, as well as on their finances [12]. Home care services was another source of support highlighted in this review, however, the use of these services was associated with some issues such as lack of continuity of care, inadequate understanding of the needs of older adults, as well as lack of information on services, particularly for those without links to people with similar needs or health and social care services. Some of these issues were recognised as areas of improvement in the delivery of home care to older adults by the Care Quality Commission in the UK [82].

This review also showed that in spite of the physical challenges faced, many older adults demonstrated a desire to cope with their illness and maintain independence. This was demonstrated in developing self-care strategies, using mobility aids and home adaptations equipment and continuing to perform activities despite them being physically difficult. The importance of maintaining independence and supporting older adults to remain mobile and care for themselves are, indeed, well-recognised priorities to official bodies [83, 84], as well as to older adults themselves [85–88]. However, some barriers were identified in this review that might interfere with achieving this. For example, many of the self-care strategies adopted by older adults were based on their own personal experience, with clear lack of information on professional advice. Although some of these strategies can be useful, recent evidence suggest that coping strategies adopted by older adults, particularly in the area of mobility, might be inappropriate and do not address their needs [89]. Also, this review highlighted that managing multiple co-morbid conditions can be challenging and further complicated by lack of professional advice and poor coordination between services. However, evidence in this area came mostly from participants with dementia and visual impairment, suggesting the need to further understand the support required by older adults to manage different clusters of multiple conditions. Additionally, although many older adults were positive about the use of technology in facilitating their daily lives, some barriers were identified that interfered with its use such as lack of skills and information, cost of products and the device not being suitable for one of the co-morbid conditions. Some of these barriers were in line with previous research [90–92], emphasising the importance of addressing these barriers in order to increase technology adoption amongst older adults. Collectively, based on the evidence reviewed, there is a clear need to meet older adults’ needs in this area, as well as support them with evidence-based self-care strategies to maintain their independence as long as possible. This is of particular importance, given the challenges facing the informal and formal care system that are leaving many older adults with unmet needs in the of areas of self-care, domestic life and mobility [9].

### Psychological health

Many older adults in this review experienced a range of emotional difficulties related to living with chronic conditions. The need to increase older adults’ access to mental health support is well acknowledged in recent reports [93–96], particularly in the case of older adults with chronic conditions [94]. However, efforts to achieve this might be hindered by poor detection of mental health problems in this population [93, 94, 96], attributed in some cases to the presence of symptoms common to both physical and mental health problems such as fatigue [97], as well as lack of awareness of mental health problems amongst health professionals and older adults themselves [93, 94, 96]. In this review, mental health problems were reported or measured in a few studies only, in spite of the range of negative emotions mentioned by many participants. Further, and aside from support provided by social networks, there was a clear lack of information on formal support provided to older adults to cope with difficulties faced. Many also developed their own strategies to cope with their emotional difficulties, with limited details provided on how these strategies were developed and whether formal guidance was received. Collectively, this would suggest the need to increase older adults’ access to psychological support to cope with emotional and psychological difficulties caused or exacerbated by chronic conditions, while acknowledging that this problem might be undetected in this population.

### Other supports needed

It is also important to acknowledge that this review highlighted other areas that older adults might require support with.

*Work-* This review highlighted the value of work to some older adults. The benefits of work to older adults and society as well as the importance of supporting work in later life have been acknowledged in several recent reports [98–101]. However, this review highlighted that many older adults are still leaving work due to ill-health and lack of support from employers. This finding is in line with recent output from the Department of Work and Pensions [101] reporting that despite most employers acknowledging the importance of older workers, few took practical steps to support them. It is noteworthy that findings in this area came from grey literature and younger participants, highlighting a gap in published literature about the experiences of older participants. Understanding the support required by this population might be of particular importance, given the fact that many older adults with chronic conditions stop working years before pension age (65-years old), in spite of their preference to work beyond that [101].

*Caring-*This review also highlighted that despite the increasing number of older carers in the UK [2], there is still limited insight about the experiences of this group. Caring responsibilities can be associated with physical, mental and social challenges [2] that can complicate existing difficulties related to chronic conditions, highlighting the need to further understand the support required by this population.

This review resulted in some implications for future research and work around the care and support needs of older people. It highlighted the importance of taking into consideration the needs of older people when designing services or solutions targeting them, as many available support services do not cater to their needs such as care services and technology products. It also highlighted gaps in the knowledge that future research needs to consider: 1) understand the strategies used by older people to cope with social difficulties; 2) understand the support required by older people to manage various clusters of multiple morbidities; 3) understand how to better detect the psychological needs in the older population; and 4) understand the care and support needs of older carers and older workers. Findings of this review will also be shared with older adults to validate the experiences and views that were expressed in this review, as well as to identify priority areas for care and support.

#### Strengths and limitations

One of the main strengths of this synthesis is the broader view taken when identifying the care and support needs of older adults living at home with chronic conditions. Understanding the physical, social, psychological challenges as well as the wider context in which older adults live and interact is pivotal to designing effective solutions and increasing the adoption of these solutions. Also, it is important to acknowledge that the aim was not to map the individual needs, as these are highly dependent on the interactions between the individual’s intrinsic and extrinsic environment [27], but to understand areas where older adults might need care and support. Hence, the views of participants in this review might not necessarily reflect the experiences of older adults with similar conditions and living circumstances. Another strength of this review is the use of the ICF framework, which offered an opportunity to use standard language understood nationally and internationally [27, 28]. The use of ICF also facilitated the categorisation of the environmental factors, which otherwise would have been challenging.

There are also some limitations that need to be acknowledged. Grey literature was an important additional source to this review, however, given the nature of search in the grey literature, there is a possibility that a key reference or article was missed. Another limitation is that the screening of full articles and grey literature, as well as the data synthesis and interpretation were conducted by the primary author (SA). There is a possibility that the screening process, the analysis and interpretation of the themes was influenced by the author’s own perceptions or understanding of the topic. However, an opinion from a second reviewer was sought during the process of grey literature and full articles screening in case of uncertainty, and also the themes synthesis and interpretation were discussed regularly with the research team to reduce potential bias. Also, no restriction was made on the study design or quality, since the scoping review is meant to scope evidence in the area under investigation. However, there is a chance that the variations in the study designs and quality affected the final synthesis.

## Conclusions

In summary, this review provided an overview of the areas that older adults living at home with chronic conditions in the UK might need care and support with. It was clear from the evidence reviewed that older adults living with chronic conditions are faced with some challenges in their social lives, psychological health, and activities related to self-care, domestic lives and mobility. It was also clear that despite these challenges older adults valued independence and demonstrated a desire to cope with their illness. However, lack of professional support and barriers associated with some services interfered with these efforts, highlighting the fact that many services and care delivery models are still not based on the needs of older adult. Thus, these findings reinforced the importance of tailoring interventions and support services that take into consideration the needs of older adults.

## Additional files

Additional file 1: Summary of the characteristics of the studies included in the final analysis. 40 articles were examined in full. The following table summarises the characteristics of these articles which include the study objective, its location, methods used and participants’ characteristics. (DOCX 39 kb)

Additional file 2: Themes and sub-themes identified in studies grouped by conditions. The following table list the studies that reported or discussed each theme and sub-theme grouped by conditions. (DOCX 21 kb)

Additional file 3: Summary of the findings of each of the themes based on the ICF framework. The following table summarises the findings of each of the themes based on the ICF framework and coding system- a) body functions, b) activities and participation, c) environmental factors. (DOCX 30 kb)

## Data Availability

The datasets used and/or analysed during the current study are available from the corresponding author upon request.

## Abbreviations

ADL: Activities of Daily Living
DM: Dementia
HF: Heart failure
ICF: International Classification of Functioning, Disability and Health
PCC framework: Population, Concept and Context framework
UI: Urinary Incontinence
UK: United Kingdom
WHO: World Health Organisation; Disability and Health
