# Correction to: Understanding the care and support needs of older people: a scoping review and categorisation using the WHO international classification of functioning, disability and health framework (ICF)

**DOI:** 10.1186/s12877-019-1279-8

**Published:** 2020-01-22

**Authors:** Sarah Abdi, Alice Spann, Jacinta Borilovic, Luc de Witte, Mark Hawley

**Affiliations:** 1 Centre for Assistive Technology and Connected Healthcare, School of Health and Related Research, The Innovation Centre, The University of Sheffield, 217 Portobello, Sheffield, S1 4DP UK; 2 Aging and Health Research Unit, Faculty of Health Sciences, The University of Sydney, 75 East Street, J block, Lidcombe, NSW 2141 Australia

**Correction to: BMC Geriatr**

**https://doi.org/10.1186/s12877-019-1189-9**

Following publication of the original article [1], we have been notified that acknowledgement should be added to the text of the articles. The Acknowledgement section should read as follows:

The authors gratefully acknowledge the support of the Economic & Social Research Council (award ES/P009255/1, Sustainable Care: connecting people and systems, 2017–21, Principal Investigator Sue Yeandle, University of Sheffield). Prof Mark Hawley is a Theme Lead for the National Institute for Health Research (NIHR) Devices for Dignity MedTech Co-operative (MIC). The work of the Devices for Dignity MIC is funded by the NIHR. The views expressed are those of the authors and not necessarily those of the NHS, the NIHR or the Department of Health and Social Care.
