# Supplementary material for: Understanding the care and support needs of older people: a scoping review and categorisation using the WHO international classification of functioning, disability and health framework (ICF)
Source: BMC Geriatr. 2019 Jul 22;19:195. doi: 10.1186/s12877-019-1189-9 (PMC6647108; doi:10.1186/s12877-019-1189-9)
Supplement: Supplementary file 1 — Summary of the characteristics of the studies included in the final analysis. 40 articles were examined in full. The following table summarises the characteristics of these articles which include the study objective, its location, methods used and participants’ characteristics. (DOCX 39 kb) [file 12877_2019_1189_MOESM1_ESM.docx]

**Additional file 1.** Summary of the characteristics of the studies included in the final analysis

| **Published articles** | | | | | | |
| --- | --- | --- | --- | --- | --- | --- |
| **No.** | **Source** | **Location** | **Study aim(s)** | **Condition under investigation** | **Methods** | **Participants characteristics** |
| 29 | (Sørbye et al., 2009) | Maidstone and Ashford- England as part of a European project | Investigate the prevalence of Urinary Incontinence and the need for assistance in a sample of home care users in Europe | Urinary Incontinence | -Cross-sectional study  -Random sample of home care users aged 65 years and above was selected  -Participants were assessed using the International Resident Assessment instrument for Home Care (RAI-HC).  -Interviews took place in participants own home  -Physical functioning was assessed using two hierarchical scales for Activity of Daily Living (ADL) and Instrumental Activity of daily Living (IADL) | N:289  Female (%): 74  Mean age (SD): 82.6 (3.3)  Living alone (%): 65  ADL dependency (%): 24  IADL dependency (%): 62  Urinary incontinence (%): 39 |
| 30 | (Lawrence, Murray and Banerjee, 2009) | South London- England | Explore the needs and experiences of older adults with visual impairment and dementia | Visual Impairment and Dem  entia | -Qualitative in-depth interviews  -Participants recruited from community, voluntary and statuary health and social care services for older adults with low vision or dementia. | N: 19  Female (%): 63  Age category (%): 65-74 (5), 75-84 (35), above 80 (55)  Living alone or sheltered housing (%): 37 (living alone 20%)  Living with family or spouse (%): 26  Other living arrangement (%): 37 |
| 31 | (Nicholson et al., 2013) | London- England | Understand the experience of older adults with changing states of frailty | Frailty | -Qualitative interviews  -Participants were identified via their intermediate care team and were purposively selected if they were regarded frail and live at home. People with Dementia were excluded  -The Free Association Narrative Interview Method and the Biographic Narrative interpretative Method were used to demonstrate the narratives of the participants | N:15  Female (%): 67  Average age (range): 89.9 (86-102)  Living alone or sheltered housing (%): 60 (living alone 53)  Living with family (%): 27  Other arrangement (%): 13 |
| 32 | (Newberry, Martin and Robbins, 2015) | Leeds- England | Explore the views of people with learning disabilities about old age and ageing | Learning disability | -Qualitative semi-structured interviews  -Participants recruited through day services and community learning disability teams  -Interpretative phenomenological analysis used for data analysis | N:7  Female (%): 42  Average age (SD): 69 ± SD  Living alone (%): 14  Other living arrangement (%): 86 |
| 33 | (Nicholson et al., 2012) | London- England | Understand the experience of older adults of living and dying with frailty | Frailty | -Longitudinal qualitative interviews  -Participants were purposively selected via their intermediate care team and were selected if they were regarded frail and live at home. People with Dementia were excluded  -The Free Association Narrative Interview Method and the Biographic Narrative interpretative Method were used to demonstrate the narratives of the participants | N:17  Female (%): 71  Age range: 86-102  Living alone or sheltered housing (%): 59%  Living with spouse or family: 41% |
| 34 | (Nyman, Innes and Heward, 2017) | Three research sites representing the northern, middle, and southern parts of England. | Explore the social care and support needs of adults living with dementia and visual impairment | Visual Impairment and dementia | -Qualitative semi-structured interviews  -Participants identified via local organisations and service providers and were recruited if they were community dwelling, received a formal diagnosis or in the process of assessment, and have visual impairment.  -Thematic analysis used for data analysis | N:26  Female (%): 65  Mean age (range): 82.1 (58-96)  Living alone (%): 50  Living with spouse or partner (%):46  Other arrangements (%): 4 |
| 35 | (Górska et al., 2013) | Midlothian, Scotland | Understand the lived experience and service related needs of people with dementia | Dementia | -Qualitative semi-structured and narrative interviews  -Participants were identified via formal and voluntary health and social care services and were selected if they had confirmed diagnosis of dementia and experience of dementia services.  -Thematic content analysis used for data analysis | N: 20  Female (%): 92  Mean age (range): 84 (77-93)  Lived within the community (%): 68  Other living arrangement (%): 32  Severity of dementia (%): Mild (40), Moderate (25), Severe (35) |
| 36 | (Ream et al., 2008) | North, Middle and South of England | Identify the unmet needs for care and support in men with prostate cancer | Prostate Cancer | -Survey study  -Participants were recruited from six NHD trusts if they had been diagnosed with prostate cancer three to twenty-four months before data collection  -The supportive care needs was assessed via 34-item questionnaire that included four dimensions: physical and daily living, psychological, sexuality, patient care and support.  -Additionally, the EuroQol EQ-5D was used to assessed participants quality of life, and the International Prostate symptom score was used to measure lower urinary tract symptoms. | N: 741 men  Age distribution (%): less than 65 (25), 65-74 (45), above 75 (30)  Ethnicity (%): White British (91) |
| 37 | (Godfrey and Townsend, 2008) | England | Explore the meaning and experience of illness and recovery in older adults | Severe illness (eg heart attack, stroke, falls and fractures, deterioration in health or mobility) | -Qualitative interviews  -Participants were purposively selected based on their use of intermediate care services across the spectrum for old age.  -Grounded theory used for data analysis | N: 64  Mean age (range): 79 (51-94)  Female (%): 70  Living alone (%): 72  Living with spouse or family (%): 28  Ethnicity (%): White British (88) |
| 38 | (Fenlon et al., 2013) | South of England | Explore the lived experience and support needs of older women with breast cancer and co-morbidities | Breast Cancer | -Qualitative interviews and focus groups.  -Participants were identified via local organisations and libraries.  -Thematic analysis used for data analysis | N: 30 females  Age: 70 and above  Living alone (%): 33  Living with others (%): 60  Socioeconomic status (%): high or moderate deprivation areas (60)  Co-morbidities (%): 3+ (43), less than 3 (57) |
| 39 | (Walthall, Jenkinson and Boulton, 2017) | England | Explore the experience of patients with chronic heart failure of breathlessness and the impact it has on their lives | Breathlessness in Chronic Heart Failure | -Qualitative semi-structured interviews  -Participants were identified from a large tertiary care centre and were recruited if they were diagnosed with heart failure. | N:25  Female (%): 40  Average age (range, SD): 72.66 (53-86, 9.46)  Co-morbidities (%): none (44), with one or more (56) |
| 40 | (Gysels and Higginson, 2011) | London, England | Explore the lived experience of breathlessness in patients with cancer, COPD, heart failure and MND | Breathlessness in cancer, COPD, heart failure and MND | -Qualitative cross-sectional interviews and participants observation  -Participants were identified from specialist clinics at hospital, support groups and the community and were recruited if they advanced stages of the illness.  - Framework analysis was used for data analysis | N: 34  Female (%): 50  Age range: 53-84 |
| 41 | (Rabiee, 2012) | York, England | Explore the views of disabled and older people about choices of support services and how these choices relate to their perceived independence | Disability | -Qualitative longitudinal interviews  -Participants identified from specialist formal and voluntary services and were recruited if their conditions were likely to change over time, or if they experience a sudden onset of support needs | N: 50  No. of adults aged 65 and above (%): 18 (36) |
| 42 | (Hill, Dziedzic and Nio Ong, 2011) | Staffordshire, England | Explore the experience of older adults of the treatment and management of Hand Osteoarthritis | Hand Osteoarthritis | -Qualitative focus groups  -Participants were recruited if they had a formal diagnosis of hand, finger, thumb osteoarthritis, hand pain or arthralgia and presenting to primary and secondary care.  -Inductive qualitative analysis | N:28  Female (%): 84  Mean age (range): 64.9, 51-84 |
| 43 | (Martin et al., 2013) | Warwick, England | Explore the barriers to self-management faced by people with Dementia | Dementia | -Qualitative interviews  -Participants purposively recruited from charity organisation.  -Thematic content analysis was used for data analysis | N:7  Female (%): 14 |
| 44 | (Toms et al., 2015) | North Wales, England | Explore the views of people with dementia about self-management | Dementia | -Qualitative interviews  -Participants identified from memory clinic serving semi-rural population and recruited if they had early stages of dementia or above.  -Thematic analysis was used for data analysis | N:13  Female (%): 70  Mean age (range, SD): 75.4 (62-89, 8.4)  Living alone (%): 15  Living with partner (%): 85  Ethnicity (%): White British (92) |
| 45 | (Nio Ong, Jinks and Morden, 2011) | Staffordshire, England | Explore the living experience of people who are deemed to self-manage knee osteoarthritis | Chronic knee pain | -Longitudinal qualitative study, using in depth semi-structured interviews at baseline and follow up diaries collected over a 6-month period  -Participants were recruited from an existing cohort, who are participants in a longitudinal joint pain study. Participants were included in the study if they suffered from moderate to severe knee pain.  -Constant comparative method and narrative analysis was used | N:22  Female (%): 59  Age distribution (%): 50-64 (32), 65-75 (59), 75+ (9)  Living alone or sheltered accommodation (%): 23  Living with spouse (%): 77 |
| 46 | (Thurston, Thurston and McLeod, 2010) | Dundee, Scotland | Explore the socio-emotional impact of sight loss in blind and partially sighted adults | Blindness | -Mixed Methods  -Quantitative data were collected using the Mental health and Social Functioning subscales of the VRQ-25  -Qualitative data were collected using semi-structured interviews.  -Participants recruited via charity organisations  -Grounded theory used for data analysis | N: 18  Female (%): 61  Average age (range): 73.4 (45-85)  65 and above (%): 56 |
| 47 | (Stanford et al., 2009) | Manchester, England | Understand the psychosocial adjustment of patients with Age Related Macular Degeneration | Age related macular degeneration | -Qualitative methods using self-reporting diaries  -The study was part of three arms randomized controlled trial  -Participants were recruited from a specialist clinic in a hospital.  -Grounded theory framework used for data analysis | N:37  Female (%): 54  Average age (range, SD): 81.62 (73-92, 4.4)  Living alone (%): 37 |
| 48 | (Bunn et al., 2017) | South and North East of England | Explore the impact of comorbidities on service related needs for patients with dementia | Dementia | -Qualitative semi-structured interviews and focus groups  -Participants were recruited via GP practices, specialist clinics and voluntary organisations  -The majority of interviews involved the patient’s carer.  -Thematic content analysis | N:28  Female (%): 36  Average (range): 82.5 (59-94)  Living alone (%): 22  Living with spouse or family (%): 78  Co-morbidities (%): +3 (34), +2 (17)  Ethnicity (%): White British (85%) |
| 49 | (Giebel, Sutcliffe and Challis, 2015) | North West England | Explore the impaired activities at different stages of dementia | Dementia | -Secondary analysis of cross-sectional study  -Participants in the original sample were recruited via day care centres, memory clinics, community specialist teams.  -Everyday functioning was assessed via the Katz Index of Independence in Activities of daily living. Quality of life, psychopathology in dementia, depressive symptomatology and possible comorbidities were also measured. | N:122  Female (%): 71.3  Average (SD): 83 (6.5)  Living at home (%): 51.6  Severity of dementia (%): Mild (17), Moderate (46.7), Severe (36) |
| 50 | (Seidel et al., 2010) | UK | Explore activity demands and how it relates to the capability of older people in Great Britain | Disability | -Data analysis from the Disability Follow up Survey (DFS), a survey conducted by the Office for National Statistics (OFN)  -The DFS was analysed to determine the most frequent hand functions, body posture and actions in older adults with disability or long-standing illness | N: 4886  Female (%): 53.3  Age distribution (%): 55-64 (24.5), 65-84 (66.4), 85-93 (9.1)  Co-morbidities (%): none (21), 1 (27.1), 2 (26.3), 3+ (25.5) |
| 51 | (Baxter and Glendinning, 2011) | England | Explore the experience of disabled adults and older people about the use of information to choose health and social care services | Disability | -Qualitative longitudinal interviews  -Participants were identified from a formal and voluntary health and social care services and recruited if they have support needs that fluctuate, or they experience sudden onset of needs.  -Thematic analysis used for data analysis | N: 32  Female (%): 56  No. of adults aged 65 and above (%): 18 (56)  Ethnicity (%): White British (94)  Living alone (%): 50 |
| 52 | (Greenhalgh et al., 2013) | London and Manchester, England | Explore what matters to older people with assistive living needs | Multi-morbidities | -Ethnography of 40 individual cases  -Participants were recruited from a diverse ethnic and socio-economic diversity for a project on assistive technologies for healthy living in elders  -Constant comparative method | N: 40  Female (%): 67.5  Median age (range): 81 (60-98)  Ethnicity (%): White British (60), Caribbean (12.5), Asian (10), Chinese (7.5), African (5)  Living alone (%): 45  Living with partner or with others (%): 55  Almost all participants had multiple morbid conditions |
| 53 | (Burt et al., 2009) | England | Explore the palliative care experience of older adults dying from cancer and non-cancer reasons | Cancer (non-cancer conditions not specified) | -A survey study  -Participants random sampling was conducted by the Office for National Statistics (ONS).  -Bereaved carers were interviewed about the experience of older adults during the last three months of their lives. | N: 939, non-cancer (650), cancer (289)  Female (%): non-cancer (62.2), cancer (48.4)  Age distribution (%): 65-69 (5.8 non-cancer, 14.5 cancer), 70-79 (24.3 non-cancer, 43.3 cancer), 80+ (70 non-cancer, 42.2 cancer) |
| 54 | (Gardiner et al., 2009) | Leeds, England | Explore the needs of patients with Advanced Chronic obstructive pulmonary disease | Advanced COPD | -Qualitative semi-structured interviews  -Participants were recruited from primary care who participated in a pilot study that aimed to explore the palliative care needs in COPD  -Thematic analysis used for data analysis | N: 21  Female (%): 38.1  Average age (SD): 70.3 (7.5)  Age distribution (%): 50-60 (19), 61-70 (38.1), 71-80 (38.1)  Living alone (%): 28.6  Living with others (%): 71.4  100% of the sample had co-morbidities |
| 55 | (Pinnock et al., 2011) | E Lothian, Tayside, and Forth Valley, Scotland | Explore the end of life needs of patients with COPD | Advanced COPD | -Qualitative longitudinal interviews  -Participants were identified from primary and secondary clinicians in general practice and were recruited if they had end stage COPD  -Thematic narrative approach used for data analysis | N: 21  Female (%): 67%  Average age (range, SD): 71 (50-83, 8)  Comorbid disease (%): +1 (90.4) |
| 56 | (Gott et al., 2008) | Urban and Rural settings in the north and south of the UK | Explore the palliative care needs of older people with heart failure | Heart failure | -Qualitative interviews  -Participants were identified from sixteen general practice, and were recruited if they had self-reported stage 2-4 heart failure  -Thematic analysis used for data analysis | N: 40  Female (%): 48  Age distribution (%): 60-75 (57%), above 75 (43)  Living alone (%): 48  Living with others (%): 52  Socio-economic (I and II) %: 25  Socio-economic (III and IV) %: 75 |
| 57 | (Saleem et al., 2013) | London, UK | Assess the palliative care needs of patients with advanced Parkinsonism | Advanced Parkinsonism | -A cross-sectional study  -Participants were identified from a specialist clinic in one hospital and were recruited if they had advanced disease stage  -Assessment of care needs was assessed using validated the Palliative Outcome Scale Parkinson Disease (POS-PD) | N: 82  Female (%): 45.1  Average age (range, SD): 67.06 (38-86, 8.8)  Ethnicity (%): White British (78), White Irish (2.4), Asian (8.5), Black Caribbean (3.7), Chinese (1.2), others (6.1) |
| 58 | (Miranda-Castillo, Woods and Orrell, 2010) | East London, England | Identify the needs of people with dementia living alone and compare it with ones living with others | Dementia | -Qualitative semi-structured interviews  -Participants were identified from NHS facilities and voluntary organisations and were recruited if they were aged 60 and above and had a diagnosis of dementia and were living at home.  -Care needs were assessed using the Camberwell Assessment of Need for the Elderly (CANE), which covers 24 areas of physical, social, psychological and environmental needs | N: 152, living alone (28), living with others (124)  Female (%): living alone (76%), living with others (39.2)  Average age (SD): living alone 81.7(5.9), living with others 78 (6.9) |
| 59 | (Griffiths et al., 2015) | West Midlands of the UK. | Explore the lived experience of people with hip fracture | Hip fractures | -Qualitative interviews  -Participants were recruited from an existing cohort study, who were originally recruited from a single major trauma centre because of hip fracture  -Thematic analysis and cross-case analysis | N: 31  Female (%): 64.5  Average age (range): 81.5 (61-96) |
| 60 | (Stewart and McVittie, 2011) | Scotland | Understand the experience of people post fall | Falls | -Qualitative semi-structured interviews  -Participants were identified and recruited by their community physiotherapist, if they had sustained a serious fall or had falls which require medical treatment  -Interpretative phenomenological analysis for data analysis | N: 8  Female (%): 87.5  Average age (range): 84 (67-89)  Living alone (%): 87.5  All participants lived in lower socio-economic area |
| **Grey literature** | | | | | | |
|  | **Title of the document** | **Issuing organisation and date of publication** | **Type and purpose of the document** | **Condition under investigation** | **Methods** | **Sample Characteristics** |
| 61 | Understanding the lives of older people living with frailty – a qualitative investigation | IPSOS for Age UK (2014) | -Report  - Explore the lived experience of older adults with frailty | Frailty | -Qualitative ethnographic interviews followed by in-depth interviews as well as focus groups  - Potential participants contacted via contacts they knew | N:10  Female (%): 80  Average age (range): 82.9 (68 - 92)  Living alone (%): 20  Living with spouse or family (%): 80  Ethnicity (%): White British (80), Caribbean (20) |
| 62 | I am still me- a narrative for coordinate support for older people | National Voices, Age UK and UCL partners (2014) | -Report  -Understand the views of older people with frailty and how they want coordinated care to support them | Frailty | -Qualitative semi-structured interviews  - Participants were recruited purposively from Age UK day centres, hospital wards, house-bound GP patients and Age UK befriending services | N: 74  Female (%): not mentioned  Average age: 84  Ethnicity: White British (80), Indian (7), Black (6), Other (7)  Living alone (%): 63  Living with family (%): 18 |
| 63 | Behind the headlines: the battle to get care at home | Age UK (2018a) | -Report  -Explore the experience of older adults of home care services | A range of chronic conditions | -Summary of queries received by the advice centre of Age UK about social care | N: 30 cases included in the report  Female (%): 53 |
| 64 | Older people’s experience of emergency hospital readmission | Age UK (2012) | -Report  -Understand the experiences of older people with emergency hospital readmission | A range of chronic conditions | -Qualitative interviews  -Participants recruited via voluntary sector organisations | N:18  Average age: 75  Female (%): 72  Living alone (%): 78  Ethnicity (%): 33% from unspecified ethnic minority |
| 65 | Painful Journeys- why getting to hospital appointments is a major issue for older people | Age UK (2018b) | -In-depth policy report  -Understand older people’s experiences of travelling to hospital for non-emergency appointments | A range of chronic conditions | -Summary report based on data from focus groups discussion, nationally representative survey with people aged 65 and above, case studies from Age UK advice centre, survey among Age UK supporters | N: 5022 from the survey data, the number of case studies and participants in the focus group discussions was not mentioned. |
| 66 | Primary research with practitioners and people with lived experience – to understand the role of home adaptations in improving later life | Centre for Ageing Better (2018a) | -Report  -Understand the lived experience of older adults who use home adaptations | A range of chronic conditions | -Two-phase qualitative semi-structured interviews (using camera in the 1^st^ interview)  -Participants were recruited purposively via local councils and voluntary organisations  -Content analysis | N: 30  Female (%): 57  Age distribution (%): 65-74 (23), 75-84 (50), 85+ (27)  Ethnicity (%): White British (93) |
| 67 | Later Life in 2015: An analysis of the views and experiences of people aged 50 and over | Centre for Ageing Better (2015) | Explore the views and experiences of people aged 50 and over of later life | Chronic conditions- unspecified | -A multiple methods research  -6 segments of older adults were established from the English Longitudinal Study of Ageing (ELSA)  -Quantitative data was collected via survey  -Qualitative data was collected via case studies and interviews  -Participants for the case studies were recruited from eight locations in England to reflect the segment | N: Case studies (24), Interviews (12), survey (1389)  Age distribution (%): % of the survey sample were in their 60s or above  Female (%): 71 % of the case studies were females |
| 68 | Health warning for employers supporting older workers with health conditions | Centre for Ageing Better (2018b) | Understand the experiences of employees, including older workers, in managing a health condition at work | A range of chronic conditions | -A mixed methods research.  -Quantitative data was collected via an online survey  -Qualitative data was collected via focus groups and interviews  -Participants were recruited if they live or work in Greater Manchester and aged 50 and above | N: 1008 participants in the online survey, 14 participants in two focus groups (one focused on musculoskeletal conditions, and one on respiratory breathing conditions), and 8 interviews. |
