# Supplementary material for: Understanding the care and support needs of older people: a scoping review and categorisation using the WHO international classification of functioning, disability and health framework (ICF)
Source: BMC Geriatr. 2019 Jul 22;19:195. doi: 10.1186/s12877-019-1189-9 (PMC6647108; doi:10.1186/s12877-019-1189-9)
Supplement: Supplementary file 2 — Themes and sub-themes identified in studies grouped by conditions. The following table list the studies that reported or discussed each theme and sub-theme grouped by conditions. (DOCX 21 kb) [file 12877_2019_1189_MOESM2_ESM.docx]

**Additional file 2.** Themes and sub-themes identified in studies grouped by conditions

|  | **DM [34,43, 44, 49, 58]** | **DM and comorbidities [30, 34, 48]** | **Breathlessness in HF and other chronic conditions [39, 40, 52]** | **Frailty [31,33]** | **Falls and Fractures [59, 60]** | **Osteoarthritis [42, 45]** | **Cancer [36,38]** | **Sight loss [46, 47]** | **UI [29]** | **Disability [32, 41, 50, 51]** | **Multiple chronic conditions [52]** | **Severe illness, unspecified [37]** | **End of life conditions (cancer, COPD, HF Parkinson) [53, 54-57]** | **Grey Literature reports**  **[61-67]** |
| --- | --- | --- | --- | --- | --- | --- | --- | --- | --- | --- | --- | --- | --- | --- |
| **Body functions** |  |  |  |  |  |  |  |  |  |  |  |  |  |  |
| - Impaired mental functions | **X** [43, 44, 49, 58] | **X** [30,34] | **X** [39, 40] | **X** [31,33] | **X** [59, 60] |  | **X** [36,38] | **X** [46, 47] |  | **X** [32] | **X** | **X** | **X** [54,56, 57] | **X** [64] |
| - Impaired physical functions | **X** [58] | **X** [34] | **X** [39, 40, 52] | **X** [31] | **X** [59] |  | **X** [36, 38] | **X** [46, 47] | **X** |  | **X** | **X** | **X** [53, 55, 57] | **X** [61, 63, 65, 66] |
| **Activities and Participation** |  |  |  |  |  |  |  |  |  |  |  |  |  |  |
| - Mobility |  | **X** [30] | **X** [39] | **X** [33] | **X** [59] | **X** [45] | **X** [36] | **X** [47] |  | **X** [50] |  | **X** | **X** [57] | **X** [61, 63, 66] |
| - Self-care | **X** [43, 44, 49, 59] |  | **X** [39,40] | **X** [31] | **X** [59] | **X** [45] | **X** [36, 38] |  | **X** |  |  | **X** | **X** [52] | **X** [61, 63] |
| - Domestic life | **X** [58] |  | **X** [39] |  |  |  |  | **X** [47] | **X** | **X** [50] | **X** | **X** | **X** [52] | **X** [61, 63] |
| - Interpersonal interactions and relationships, community and social life | **X** [44, 58] | **X** [30,34] | **X** [39, 40] | **X** [31,33] |  | **X** [45] |  | **X** [47, 48] |  | **X** [32] | **X** | **X** | **X** [52] | **X** [61, 62, 67, 68] |
| **Environmental factors** |  |  |  |  |  |  |  |  |  |  |  |  |  |  |
| - Relationships with family, friends, community and their attitude | **X** [44, 58] | **X** [30, 48] | **X** [40] | **X** [33] | **X** [59] | **X** [45] | **X** [36] | **X** [47, 48] | **X** | **X** [51, 57] | **X** | **X** | **X** [56, 57] | **X** [61, 62, 65, 66, 67] |
| - Relationships with professionals and attitude | **X** [35, 44] | **X** [34, 48] | **X** [40] | **X** [31] | **X** [59, 60] | **X** [45] | **X** [38] | **X** [47] | **X** | **X** [51] | **X** |  | **X** [54, 55, 56, 57] | **X** [61, 63, 64, 65] |
| - Services (eg. Health services, social care and transportation) | **X** [35, 44, 58] | **X** [30, 48] | **X** [ 40, 52] | **X** [33] |  |  | **X** [36, 38] | **X** [47, 48] |  | **X** [51] |  | **X** | **X** [55] | **X** [61, 63, 65] |
| - Product and technology | **X** [44] | **X** [34] | **X** [39] | **X** [31] | **X** [59] | **X** [42, 45] | **X** [36] | **X** [47] |  | **X** [41] | **X** | **X** |  | **X** [61, 66] |

HF: Heart failure, DM: Dementia, UI: Urinary Incontinence
