# Supplementary material for: Understanding the care and support needs of older people: a scoping review and categorisation using the WHO international classification of functioning, disability and health framework (ICF)
Source: BMC Geriatr. 2019 Jul 22;19:195. doi: 10.1186/s12877-019-1189-9 (PMC6647108; doi:10.1186/s12877-019-1189-9)
Supplement: Supplementary file 3 — Summary of the findings of each of the themes based on the ICF framework. The following table summarises the findings of each of the themes based on the ICF framework and coding system- a) body functions, b) activities and participation, c) environmental factors. (DOCX 30 kb) [file 12877_2019_1189_MOESM3_ESM.docx]

**Additional file 3.** Summary of the findings of each of the themes based on the ICF framework and coding system- a) body functions, b) activities and participation, c) environmental factors

1. Body functions

| **Impaired specific mental functions** | **References** | **ICF code** | **Impaired physical functions** | **References** | **ICF code** |
| --- | --- | --- | --- | --- | --- |
| Range of negative emotions | [30, 32, 36, 37, 39, 40, 43, 44, 48, 54, 56, 57, 59, 60, 64] | b1522 | Sensation of pain | [31, 36, 37, 39, 40, 52, 53, 56, 57, 61, 63, 65, 66, 68] | b280 |
| Range of negative emotions, unspecified | [33, 36, 38, 49, 58, 59, 68] | b1529 | Impaired seeing functions | [30, 34, 46, 47, 48, 58, 65] | b210 |
| Loss of identity | [31, 37, 43, 48, 60] | b180 | Fatigability | [31, 37, 40, 52, 57, 61] | b4552 |
| Negative outlook | [37, 39, 43, 47] | b1265 | Breathlessness | [39, 40, 52, 53, 55] | b440 |
| Loss of confidence | [33, 44, 60, 52] | b1266 | Urinary incontinence | [36, 58, 63, 66] | b6202 |
| Impaired attention functions | [34, 44, 52] | b140 | Impaired functions related to the digestive system | [36, 53, 57] | b510- b599 |
| Impaired memory functions | [34, 44] | b144 | Impaired hearing functions | [34, 58] | b230 |
| Poor motivation | [39, 44] | b1301 | Impaired mobility of joints | [38, 52] | b710 |
| Impaired expression of language | [44] | b1671 | Impaired movement function (falls) | [57] | b789 |
| Impaired sleep functions | [57] | b134 | Impaired sexual functions | [36] | b640 |
| Poor cognitive functions | [52] | b164 |  |  |  |
| Impaired orientation to place | [30] | b1142 |  |  |  |
| Visual hallucinations | [30] | b156 |  |  |  |

1. Activities and participation- challenges faced

| **Mobility** | **References** | **ICF code** | **Self-care** | **References** | **ICF code** | **Domestic life** | **References** | **ICF code** | **Communication, interpersonal interactions and relationships, community and social life** | **References** | **ICF code** |
| --- | --- | --- | --- | --- | --- | --- | --- | --- | --- | --- | --- |
| Walking difficulty | [33, 37, 57, 59, 61, 66] | d450 | Difficulty with washing oneself | [39, 49, 60, 63, 66] | d510 | Difficulty with household tasks (e.g. Cleaning, hovering etc.) | [37, 39, 48, 50, 52, 53] | d640 | Social isolation and loneliness | [30, 31, 33, 34, 37, 39, 40, 47, 48, 52, 61, 62, 67, 68] | d9205 |
| Difficulty with changing body position | [39, 45, 50, 63] | d410 | Difficulty with dressing | [37,38, 49, 59, 63] | d540 | Difficulty with shopping | [37, 47, 53, 63] | d6200 | Difficulty with engaging in hobbies | [30, 39, 47, 48, 52] | d9204 |
| Difficulty with lifting and carrying objects | [38, 39] | d430 | Self-care, unspecified | [29, 38, 52, 58, 61] | d599 | Difficulty with preparing meals | [47, 53, 61] | d630 | Need to participate in voluntary and non-voluntary work | [32, 45, 67, 68] | d710 |
| Difficulty with hand and arms use | [50, 57] | d445 | Difficulty with toileting | [29, 49, 59] | d530 | Domestic life, unspecified | [29] | d699 | Lack of support at work | [67, 68] | d710 |
| Difficulty with moving around in different location | [47] | d460 | Maintaining one’s health | [43, 44] | d570 |  |  |  |  |  |  |
|  |  |  | Difficulty with eating and drinking | [63] | d550, d560 |  |  |  |  |  |  |
| Mobility problems, unspecified | [36] | d499 | Difficulty assisting others | [5] | d669 |  |  |  |  |  |  |

1. Activities and participation- strategies used to manage own physical and mental health

| **Self-care- looking after one’s health d570** | **References** | **ICF code** |
| --- | --- | --- |
| **Physical health** | | |
| Establishing a daily routine | [31, 33, 37, 44, 66] | d5702 |
| Ensuring one’s physical comfort through pacing oneself and changing the body movement to a comfortable position | [31,45,61] | d5700 |
| Managing diet and keeping physical active | [67] | d5701 |
| Attending pulmonary classes to manage breathlessness | [40] | d5702 |
| Using alternative therapy for hand osteoarthritis | [42] | d5702 |
| **Mental health** | | |
| Accepting limitations caused by long-term conditions | [30, 37, 39, 44, 47, 53, 55, 60] | d5702 |
| Adopting positive outlook | [44, 47, 57, 60] | d5702 |
| Occupying time with activities | [35, 39, 45] | d5702 |
| Spirituality | [40, 47, 61] | d5702 |
| Humour | [39, 61] | d5702 |
| Denial | [40, 47] | d5702 |

1. Environmental factors

| **Family relationships (e310-e325) and attitude (e410-460)** | **References** | **Professional relationships (e355-e360) and attitude (e410-460)** | **References** | **Services, systems and policies (e5408, e5600, e5750, e5800, e5250)** | **References** | **Products and technology (e115- e155)** | **References** |
| --- | --- | --- | --- | --- | --- | --- | --- |
| **Facilitators** | | | | | | | |
| Family and friends are important support system | [29,30, 33, 36, 37, 44, 45, 47, 48, 51- 53, 57, 58, 59, 61, 62, 65-67] | Care professionals are important support system | [31, 35, 38, 44,47, 48, 51, 52, 54, 55, 59- 61, 63- 65] | Positive feedback about specialist services | [37,44, 47, 61] | Use products and technology for daily living e.g. visual, memory aids and pendants alarms | [34, 37, 41, 42, 44, 45, 47, 52, 59, 60, 61, 66] |
| Bring joy, happiness, pleasure, companionship to participants’ lives | [30, 32, 45, 47, 52, 58, 61, 67] | Health professionals, particularly specialist nurses, are primary and trusted source of information | [38, 51, 54, 55, 64] | Home care is important support system | [29, 37, 58, 59], | Use aids for indoor and outside mobility | [34, 37, 45, 59, 66] |
| Assist with daily activities and facilitate being at home | [29, 33, 58,59, 61] | A source of comfort and reassurance | [31, 52, 60] | Written information from official bodies is useful source | [38, 51] | Adapting home | [33, 34, 41, 59, 66] |
| They navigate the system and find information on participants’ behalf | [47, 48, 51] | Assist with self-management | [44] |  |  | Tailoring adaptations to participants’ needs | [66] |
| **Barriers** | | | | | | | |
| Patronizing attitude | [30, 44, 48] | Lack of information and advice on conditions  Or co-morbid conditions  Or care after hospital | [35, 40, 42, 48, 54, 56, 57, 63]  [38, 48, 61]  [64] | Lack of communication, coordination and integration between services | [30, 35, 40, 44, 48, 64] | Negative perception of technology or equipment | [31, 52, 60 61] |
| Stigma by the society | [40, 44] | Feel undermined by health and social care professionals and not listened to | [31, 35, 60, 61] | Lack of services | [35, 44, 53, 61, 63, 65] | Cost of products | [34, 41, 66] |
| Unmet needs in areas supported by family carers | [58, 61] | Lack of continuity of care | [34, 35, 63] | Lack of information on care services and care pathways | [35, 44, 51, 63, 65, 66] | Uncomfortable and not useful | [36, 47] |
| Not feeling understood by family and friends | [48] | Formal carers disturb participants’ routine | [33] | Unresponsive social care services | [63, 66] | Lack of information | [41, 42] |
| Carers have different priorities compared to participants | [52] | Care workers not trained | [63] | Not satisfied with information from internet or leaflet | [51] | Devices not suitable for one of the comorbid conditions | [34] |
| Carers’ fears limit mobility | [59] |  |  | Poor housing | [55] | Interfere with daily lives | [52] |
|  |  |  |  | Poor access to accessible, comfortable and reliable transport services | [65] | Not taking medication due to side effect | [42] |
|  |  |  |  |  |  | Poorly fitted or unattractive equipment | [66] |
|  |  |  |  |  |  | Difficulty to learn skills | [34] |
